# Neuregulin‐1 alleviate oxidative stress and mitigate inflammation by suppressing NOX4 and NLRP3/caspase‐1 in myocardial ischaemia‐reperfusion injury

**DOI:** 10.1111/jcmm.16287

**Published:** 2021-01-20

**Authors:** Fuhua Wang, Huan Wang, Xuejing Liu, Haiyi Yu, Xiaomin Huang, Wei Huang, Guisong Wang

**Affiliations:** ^1^ Department of Critical Care Medicine The Affiliated Hospital of Qingdao University Qingdao China; ^2^ Department of Cardiology Peking University Third Hospital Key Laboratory of Cardiovascular Molecular Biology and Regulatory Peptides Ministry of Health Key Laboratory of Molecular Cardiovascular Sciences Ministry of Education, Beijing Key Laboratory of Cardiovascular Receptors Research Beijing China; ^3^ Experimental and Translational Research Center Beijing Friendship Hospital Capital Medical University Beijing China; ^4^ Department of Fundamental Oncology University of Lausanne Lausanne Switzerland; ^5^ Ludwig Institute for Cancer Research University of Lausanne Epalinges Switzerland; ^6^ The Seventh Affiliated Hospital of Sun Yat‐Sen University Shenzhen China; ^7^ Institute of Cardiovascular Sciences and Key Laboratory of Molecular Cardiovascular Sciences Ministry of Education Peking University Health Science Center Beijing China

**Keywords:** ERK1/2, Neuregulin‐1, NLRP3, NOX4

## Abstract

Neuregulin‐1 (NRG‐1) is reported to be cardioprotective through the extracellular‐regulated protein kinase (ERK) 1/2 pathway in myocardial ischaemia‐reperfusion injury (MIRI). NOX4‐induced ROS activated NLRP3 inflammasome and exacerbates MIRI. This study aims to investigate whether NRG‐1 can suppress NOX4 by ERK1/2 and consequently inhibit the NLRP3/caspase‐1 signal in MIRI. The myocardial infarct size (IS) was measured by TTC‐Evans blue staining. Immunohistochemical staining, real‐time quantitative PCR (RT‐qPCR) and Western blotting were used for detection of the factors, such as NOX4, ERK1/2, NLRP3, caspase‐1 and IL‐1β .The IS in the NRG‐1 (3 μg/kg, intravenous) group was lower than that in the IR group. Immunohistochemical analysis revealed NRG‐1 decreased 4HNE and NOX4. The RT‐qPCR and Western blot analyses revealed that NRG‐1 mitigated the IR‐induced up‐regulation of NOX4 and ROS production. Compared with the IR group, the NRG‐1 group exhibited a higher level of P‐ERK1/2 and a lower level of NLRP3. In the Langendorff model, PD98059 inhibited ERK1/2 and up‐regulated the expression of NOX4, NLRP3, caspase‐1 and IL‐1β, which exacerbated oxidative stress and inflammation. In conclusion, NRG‐1 can reduce ROS production by inhibiting NOX4 through ERK1/2 and inhibit the NLRP3/caspase‐1 pathway to attenuate myocardial oxidative damage and inflammation in MIRI.

## 1 INTRODUCTION

Various studies have reported that inflammasomes are involved in the pathophysiology of myocardial ischaemia‐reperfusion injury (MIRI).^1^ Recently, NLRP3 inflammasome was demonstrated to be up‐regulated in MIRI.^2^ Reactive oxygen species (ROS) stimulate inflammasome during the pathogenesis of MIRI.^1^ All known NLRP3 activators generate ROS, whereas the inhibitors of ROS suppress inflammasome activation.^3^, ^4^, ^5^ NADPH oxidase (NOX) 4‐mediated ROS production promotes NLRP3 inflammasome activation.^6^, ^7^ Additionally, NOX4, which is the major ROS synthesis enzyme in the cardiac tissue, plays an important role in the cardiomyocytes.^8^ The inhibition of NOX4 activity can down‐regulate ROS production and alleviate MIRI.^9^

Neuregulin‐1 (NRG‐1), a member of the epidermal growth factor family,^10^ is expressed in Coronary microvascular endothelial cells (CMEC) and exerts a paracrine effect. Additionally, NRG‐1 is involved in the regulation of cardiac development ^11^ and adult cardiac function.^12^ Erythroblastic leukaemia viral oncogene homolog 4 (ErbB4), a tyrosine kinase receptor of NRG‐1, dimerizes with ErbB2 or ErbB4 upon activation by NRG‐1 ^11^ and consequently activates the downstream signalling pathways. NRG‐1 is reported to exert an antioxidant effect in the myocardial tissue and cells through the activation of the AKT/eNOS pathway.^13^, ^14^, ^15^ Previously, we had demonstrated that NRG‐1 could suppress apoptosis through ERK1/2 activation in myocardial reperfusion injury.^16^ In other models, ERK1/2 activation can suppress NOX4, which leads to the down‐regulation of ROS production.^17^ Currently, there are no studies that have examined the ability of NRG‐1 to suppress NOX4 and down‐regulate ROS production through ERK1/2 activation. In this study, we demonstrated that NRG‐1 exerts antioxidant effects by suppressing NOX4 in a rat MIRI model and a Langendorff MIRI model. Additionally, this study demonstrated that the NRG‐1‐mediated suppression of NOX4 is dependent on the activation of ERK1/2.

The role of NRG‐1 in mitigating inflammatory damage in cerebral ischaemia‐reperfusion (IR) injury has been previously reported.^18^ However, there are limited studies on the anti‐inflammatory effects of NRG‐1 in the myocardial tissue. In septic cardiomyopathy models, NRG‐1 can inhibit NLRP3 inflammasome.^6^ As NRG‐1 down‐regulated ROS production by inhibiting NOX4, this study also examined the ability of NRG‐1 to inhibit the NLRP3/caspase‐1 signalling pathway.

## 2 MATERIALS AND METHODS

### 2.1 Animals

Male adult Sprague‐Dawley rats weighing 300‐350g (6‐8 weeks old)were purchased from the Laboratory Animal Center of Peking University. We followed the Principles of Laboratory Animal Care (NIH publication no. 85‐23, revised 1996), and all the animal experimental procedure was approved by the Animal Care Committee, Peking University Health Science Center.

### 2.2 Assessment of MIRI in vivo

#### 2.2.1 MIRI model

According to previous protocols,^19^, ^20^ rats were anaesthetized by sodium pentobarbital (50 mg/kg) through intraperitoneal injection. and then a rodent respirator (ALCV9A; Shanghai Alcott Biotech Co., Ltd., Shanghai, China) was used for ventilation. The heart was exposed by a left thoracotomy through the 4th or 5th intercostal space. After the pericardium was removed, the left coronary artery (LCA) was occluded beneath the left atrial appendage by a 6‐0 silk suture for 45 minutes. Ischaemia was confirmed by the myocardium blanching, ventricular dyskinesia and elevation of ST segment on the ECG. The heart was then reperfused for 24 hours by untying the knot, and reperfusion was confirmed by a marked hyperaemic response.^21^

#### 2.2.2 In vivo experimental protocol

**FIGURE 1:**
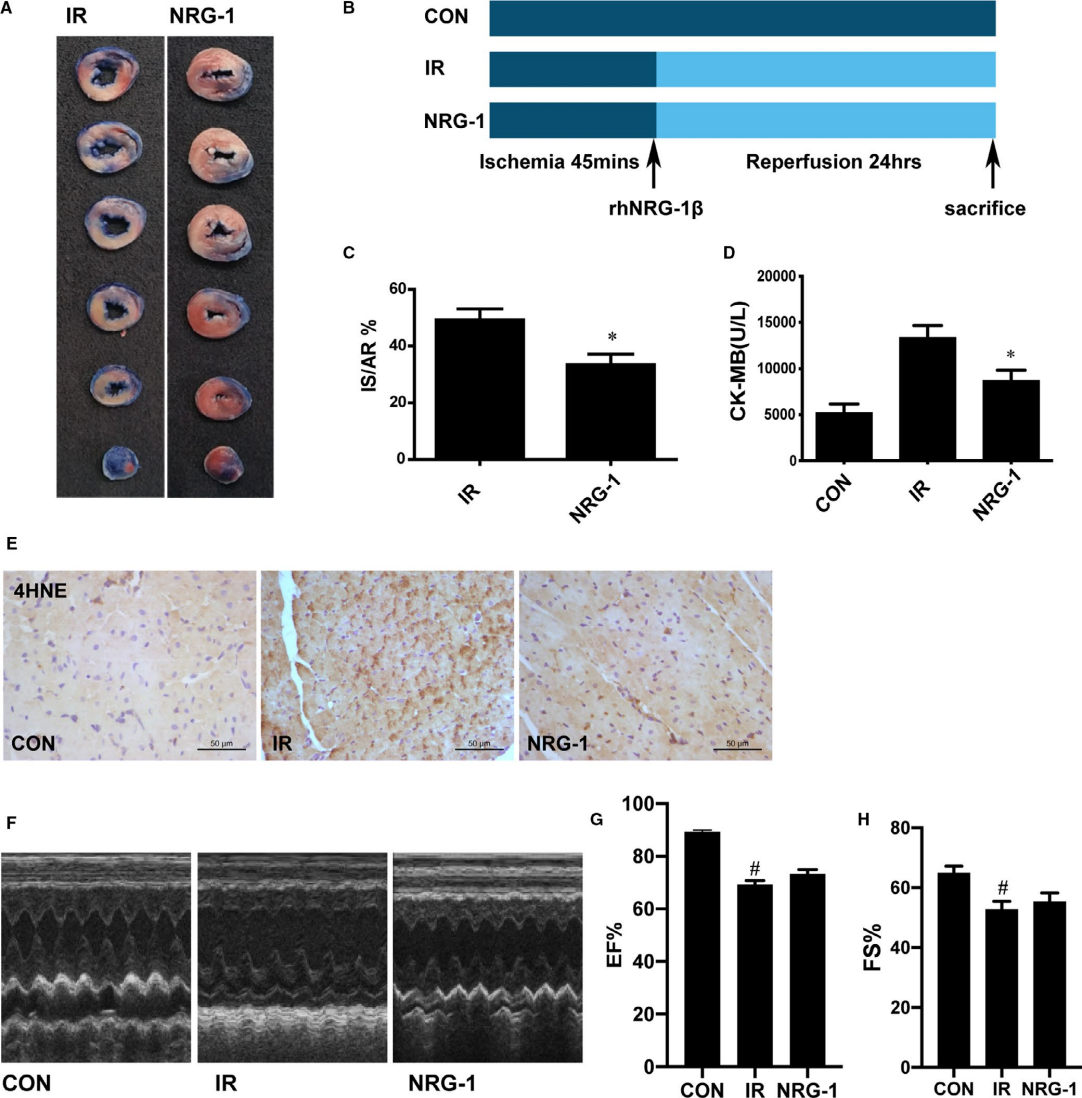
NRG‐1 protects against myocardial ischaemia‐reperfusion injury. A, Representative cardiac tissue sections stained with Evans blue and triphenyl tetrazolium chloride [blue, non‐ischaemic area; non‐blue, area at risk (AR); white, infarct size (IS)]. B, The in vivo experiment protocol. C, The ratio of IS/AR. (D) Serum creatinine kinase‐MB concentration. E, Representative paraffin‐embedded cardiac sections subjected to immunohistochemical staining to detect 4‐hydroxynonenal (4HNE) at the risk area. The cellular nuclei are shown in blue, whereas 4HNE‐positive areas are shown in brown. F, Representative M‐mode echocardiograms for each group, with (G) EF; H, FS quantified in bar graphs. Data are represented as mean ± standard error of mean (n = 6; *P < .05 vs. IR, ^#^ *P* < .05 vs. CON). CON, sham‐operated control; IR, ischaemia‐reperfusion; NRG‐1, IR + NRG‐1

The rats were divided into three groups randomly: (1) CON (control, n = 12) group, in which a left thoracotomy was done without occlusion of LCA; (2) IR (ischaemia reperfusion, n = 20, motality 40%,12 rats were used for the followed experiments) group, in which 45 minutes were for LCA occlusion and 24 hours were for reperfusion; (3) IR + NRG‐1 (n = 18, motality 33%,12 rats were used for the followed experiments) group, in which the LCA occlusion lasted for 45 minutes, and just before 24 hours’ reperfusion, recombinant human NRG‐1β2 (rhNRG‐1β2, 3 μg/kg, Prospec, Israel) was injected via the jugular vein intravenously(Figure 1B). The dosage of NRG‐1 in this study was based on our previous study.^16^

### 2.3 MIRI model in Langendorff apparatus

#### 2.3.1 Heart preparation

According to a previous protocol,^22^ we anaesthetized the rats by sodium pentobarbital (50 mg/kg) and removed the heart to a Langendorff apparatus. The heart was then perfused through the aorta retrogradely with the Krebs‐Henseleit (K‐H) buffer (NaCl 118.5 mM, KCl 4.7 mM, KH_2_PO_4_ 1.2 mM, NaHCO_3_ 25.0 mM, MgSO_4_ 1.2 mM, glucose 11 mM and CaCl_2_ 2.5 mM, at pH 7.4 and gassed with 95% O_2_ and 5% CO_2_ at 37°C). The heart was equilibrated for 20 minutes at 70 mmHg.

#### 2.3.2 Isolated heart experimental protocol

The rats were divided to four groups randomly(n = 12): (a) CON (no‐intervention) group; (b) IR group, in which the coronary flow was blocked for 30 minutes, and then, the heart was reperfused for 2hrs; (c) NRG‐1(IR + NRG‐1) group, in which 20 ng/mL NRG‐1 (the dosage of NRG‐1 was according to our previous study^16^) was perfused for 20 minutes; (d) NRG‐1 + PD(IR + NRG‐1 + PD98059, an inhibitor of ERK1/2) group, in which the treatment was similar to the NRG‐1 group, but 20 μM PD98059 (the dosage of PD was determined according to a previous study^16^) was perfused for 10 minutes before the reperfusion and lasted for 20 min at the reperfusion period. After the ischaemia period, all the hearts were reperfused for 2hrs and then were collected for the followed experiments(Figure 5C).

### 2.4 Assessment of infarct size

The staining was performed according to a previous protocol.^23^ At the end of the reperfusion in vivo, we ligated LCA again and infused the heart with Evans blue (Sigma, St. Louis, MO, USA, 0.25% in saline) from the aorta retrogradely. The non‐ischaemic part of ventricle was stained blue, the non‐stained part represented the area at risk (AR, non‐blue region). When the heart was frozen for 30 minutes at − 20°C, the heart was segmented into 6 slices of 2 mm thickness. The heart slices were incubated in 1% triphenyltetrazolium chloride (TTC, Sigma, St, Louis, MO, USA) in phosphate buffer (pH 7.4) for 10 min at 37°C and then immersed in 4% paraformaldehyde for 24 hours. Infarct Size (IS, white region) was differentiated by TTC staining from the non‐infarct AR (red region).In the experiment in vitro, the rat heart slices were single stained by TTC without Evans blue according to a previous protocol.^22^The red region indicated the non‐infarct area of the left ventricle (LV). Finally, the slices were arranged from base to apex and photographed digitally. Digital images of the heart slices were analysed by ImageJ software (NIH, USA) to measure the IS and AR region. The result is presented as IS/AR% for experiment in vivo. And in Langendorff experiment, the result is presented as IS/LV% according to a previous study.^21^

### 2.5 Western blotting

When the rat was sacrificed, the risk area of the LV was frozen by liquid nitrogen and then stored at −80°C. Frozen heart tissues for protein extraction were homogenized in RIPA solution. As previously described, 80 μg extracted protein was used for SDS‐PAGE and immunoblotting.^24^ECL chemiluminescence was performed by an imaging system (molecular imager, ChemiDoc XRS, Bio‐Rad, USA).And the bands density were detected by the same software. All protein levels were standardized to GAPDH. Primary antibodies in the present study were as follows: T‐ERK1/2, ^202^Thr/^204^Tyr‐P‐ERK1/2, NLRP3, GPX1, caspase‐1 and IL‐1β (rabbit monoclonal antibodies, Cell Signaling Technology, USA); GAPDH (mouse anti‐human monoclonal antibody, Millipore, USA); NRG‐1 and NOX4 (rabbit polyclonal antibodies, Abcam, USA) and appropriate horseradish peroxidase(HRP)‐conjugated secondary antibody (ZSGB‐BIO, China).

### 2.6 Immunohistochemistry

Tissue sections were deparaffinized and then blocked with CAS‐Block (Invitrogen, CA) for 1 h. Incubated the sections with primary antibody (NOX4, 4HNE, CD31; Abcam, USA) for 2h at 37°C followed by incubation with secondary antibody linked with HRP for 1 h at 37°C. Place sections in solution for DAB reaction for 3min. At last, sections were then stained with haematoxylin for 2 min, washed with PBS, and mounted with mounting medium (Abcam, USA). Capillary density was assessed based on the capillary/myocyte nucleus (C/M) values as previously described.^25^

### 2.7 RNA isolation and quantitative real‐time PCR

The total RNA of heart tissues was isolated using Trizol reagent (Invitrogen, USA). And a RT kit (Invitrogen, USA) was used for the production of the first‐strand cDNA. 35 cycles were designated for amplifications in the Mx3000 Multiplex Quantitative PCR System (Stratagene, USA) with SYBR green fluorescence (Molecular Probes, Eugene, USA). Each cycle included heating denaturation for 30 seconds at 94°C, annealing for 30 s at 56°C and extension for 30 seconds at 72°C. Relative quantitation of gene expression normalized to GAPDH of all the samples were quantitated by the comparative CT method as previously described.^21^ The primers in this experiment were as follows: NOX4, forward TGGCCAACGAAGGGGTTAAA and reverse CACTGAGAAGTTCAGGGCGT; GPX1, forward CAGTCCACCGTGTATGCCTT and reverse GTAAAGAGCGGGTGAGCCTT;

CAT, forward TTTTCACCGACGAGATGGCA and reverse AAGGTGTGTGAGCCATAGCC.

### 2.8 DHE staining

ROS production in left ventricular myocardium in vitro was determined by dihydroethidium (DHE, Invitrogen Molecular Probes, Eugene, USA) staining. Briefly, transverse cryosections (6μm thick) of frozen hearts were fixed with acetone for 15 minutes and then washed with 0.01%PBS 3 times before the incubation of DHE (5 μM) for 30min at room temperature. DHE fluorescence was assessed by a fluorescence microscopy.

### 2.9 TUNEL assay and immunofluorescence

The harvested hearts were fixed with 4% paraformaldehyde and embedded in paraffin. Heart sections were mounted on glass slides and then deparaffinized/hydrated for TUNEL staining. TUNEL staining was performed following the manufacturer's protocol in situ cell death detection kit (Roche Applied Science).Sections were also colabelled with primary antibody against cardiac α‐sarcomeric actin antibody (1:50; Bioss) for 1 hour at 37°C in a humidified chamber to determine presence of apoptosis in cardiac myocytes. Sections were washed and incubated with secondary antibody, and the heart sections were counterstained with DAPI to stain all nuclei present in the heart section. Sections were then examined with a confocal microscope.

### 2.10 Creatine kinase‐MB detection

At 24 h post‐reperfusion, the blood samples were collected and then centrifuged at 2000rpm for 10 min at 4˚C. CK‐MB (E006‐1‐1) levels in serum were detected according to the manufacturer's protocols (Nanjing Jiancheng Bioengineering Institute). The optical density of the tetrazolium product was determined spectrophotometrically (Molecular Devices, LLC) at a wavelength of 490 nm.

### 2.11 Echocardiography

At 24 hours post‐reperfusion, echocardiography was performed on rats anesthetized with sodium pentobarbital (25 mg/kg) as described previously^26^ (15). Measurements were obtained from short axis M‐mode images and graphically represented as EF% (left ventricular ejection fraction) and FS% (left ventricular fraction shortening).

### 2.12 Statistical analysis

All data are shown as the mean ± SEM. Statistical comparisons among the groups were performed using one‐way ANOVA followed by Neuman‐Keuls post hoc test or Tukey's post hoc test. The statistical analyses were accomplished by GraphPad Prism 8.0 (Graph Pad Software, San Diego, CA). A value of *P* ≤ .05 was considered statistically significant.

## 3 RESULTS

### 3.1 Protective effects of NRG‐1 in vivo

**FIGURE 2:**
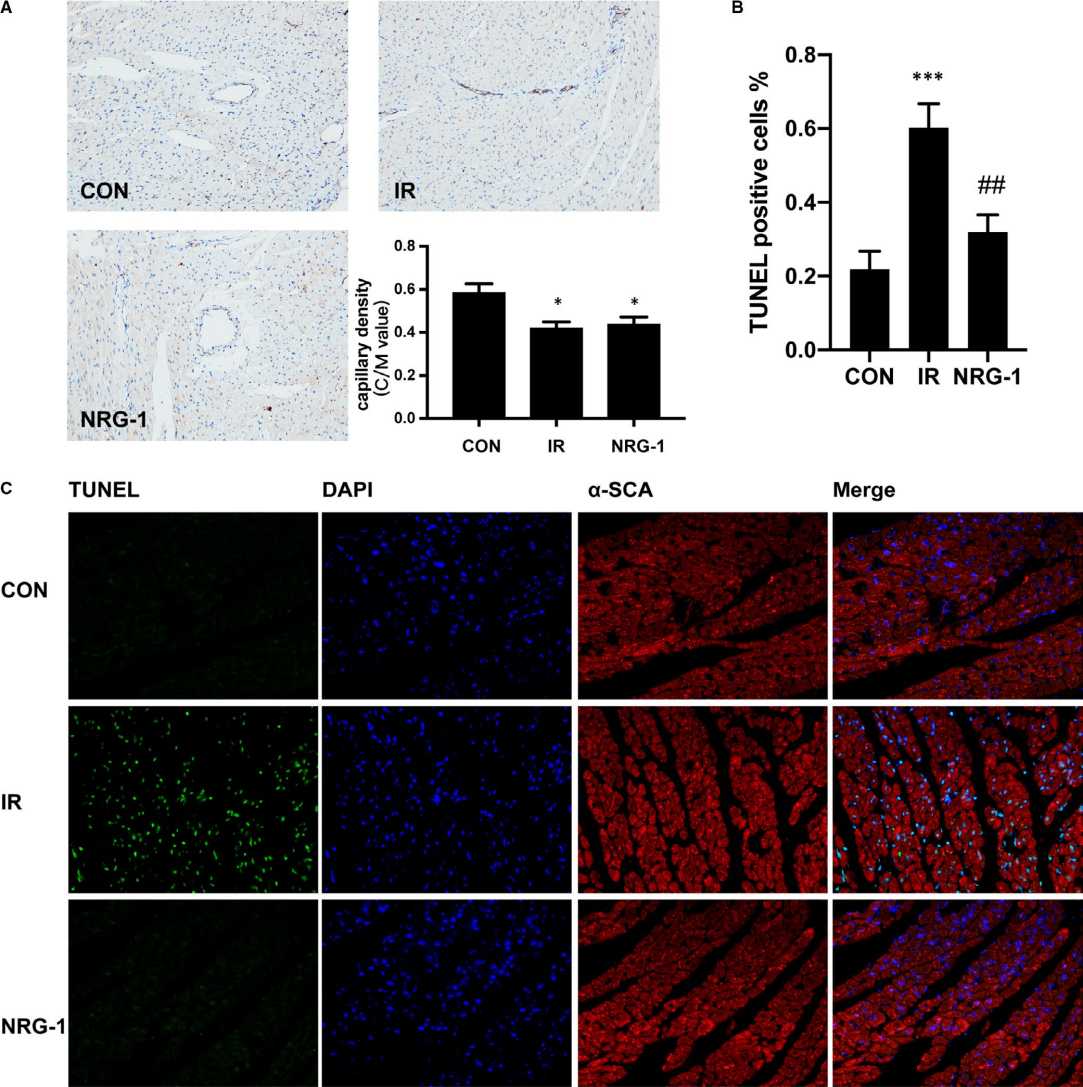
NRG‐1 shows no effect on myocardial capillary density but mitigates myocardial apoptosis. A, Immunohistochemical analysis of CD31 was performed to evaluate the myocardial capillary density. Capillary density was assessed based on the capillary/myocyte nucleus (C/M) values. Magnification: 200X. The CD31^+^ capillary endothelial cells are shown in brown. Myocardial sections were counterstained with haematoxylin (nuclei are stained blue). B, The percentage of terminal deoxynucleotidyl transferase dUTP nick end labelling (TUNEL)‐positive cells among the total number of cells. C, Representative myocardial sections subjected to the TUNEL assay. TUNEL‐positive cells are shown in green. The 4′,6‐diamidino‐2‐phenylindole (DAPI)‐stained nuclei are shown in blue. The cardiomyocytes stained with anti‐α‐SCA antibody are shown in red. Data are represented as mean ± standard error (n = 6; *P < .05 vs CON; ***P < .001 vs. CON; ##*P* < .01 vs IR). CON, sham‐operated control; IR, ischaemia‐reperfusion; NRG‐1, IR + NRG‐1

At 24 hours post‐reperfusion, Evans blue staining and TTC staining were performed to examine the IS and AR (Figure 1A). The IS/AR ratio was used as an indicator of myocardial injury.^21^ Treatment with NRG‐1 significantly decreased the IS value (Figure 1C; *P* < .05). Compared with those in the sham group, the serum CK‐MB levels were higher in the IR group and lower in the NRG‐1 group (Figure 1D; *P* < .05). The levels of 4‐hydroxynonenal (4HNE), which is the final product of lipid oxidation, are associated with the severity of oxidative damage.^27^ Immunohistochemical analysis revealed that the 4HNE level was markedly up‐regulated in the IR group (Figure 1E). However, the 4HNE levels in the NRG‐1 group were lower than those in the IR group (Figure 1E). The IR group resulted in a significant reduction in EF% (Figure 1G, *P*< .05) and FS% (Figure 1H, *P* < .05). No significant differences of EF% and FS% were found between the IR group and NRG‐1group(Figure 1F‐H).The capillary density in the IR group was significantly lower than that in the CON group (Figure 2A; *P* < .05). No significant differences were found between the IR group and NRG‐1 group (Figure 2A). Apoptosis in the IR group was significantly higher than that in the CON group (Figure 2B–C; *P* < .001). Treatment with NRG‐1 significantly mitigated apoptosis induced by myocardial reperfusion (Figure 2B; *P* < .01).

### 3.2 NRG‐1 down‐regulates the expression of NOX4 in vivo

**FIGURE 3:**
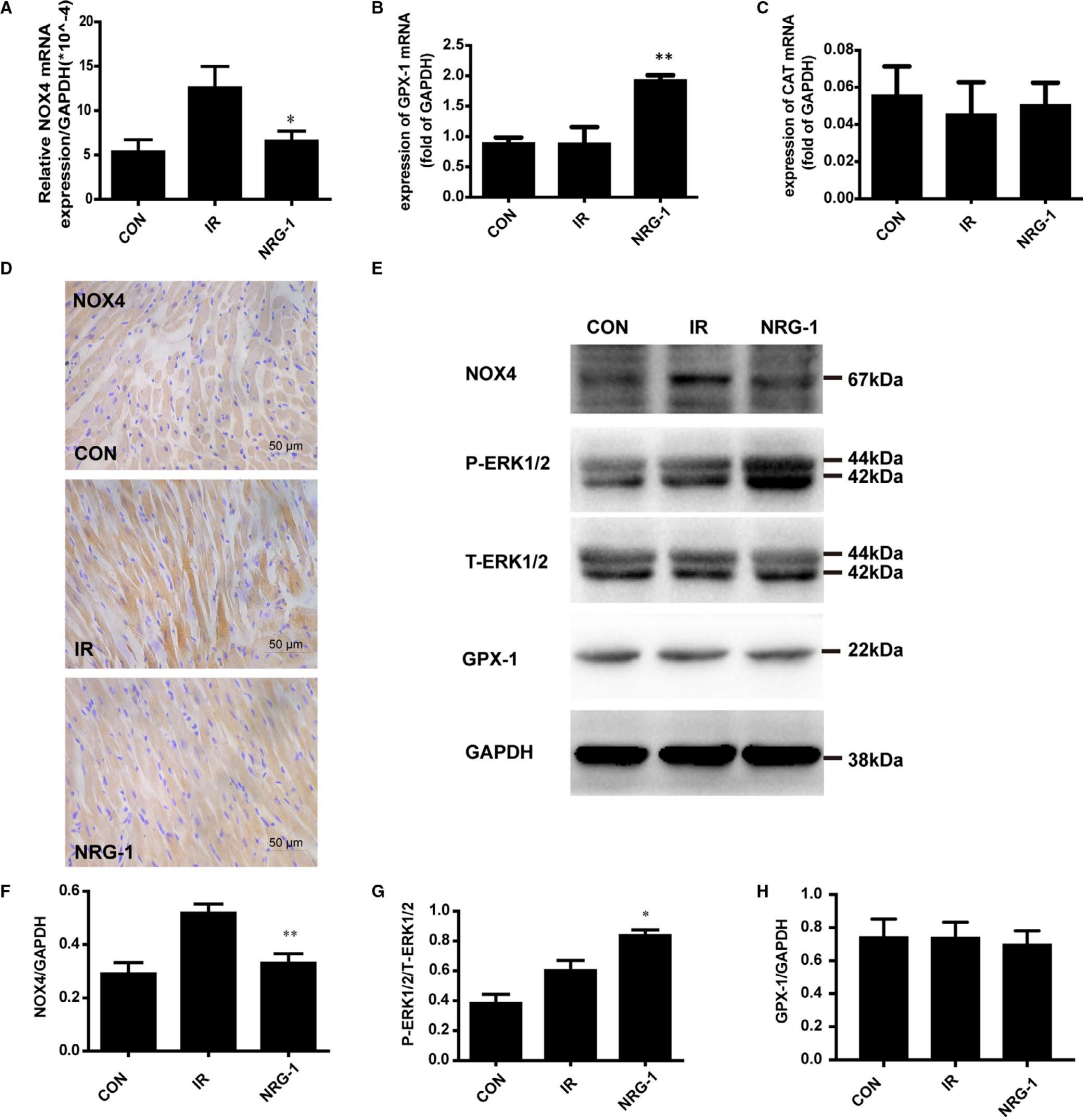
NRG‐1 down‐regulates the expression of NOX4 by activating ERK1/2. The mRNA expression levels of *Nox4* (A), *Gpx‐1* (B), and *Cat* (C) were detected using quantitative real‐time polymerase chain reaction. (D) Representative paraffin‐embedded cardiac sections subjected to immunohistochemical staining to detect NOX4 at the risk area. The cellular nuclei are shown in blue, whereas NOX4‐positive areas are shown in brown. E. Representative Western blots showing the levels of NOX4, P‐ERK1/2, T‐ERK1/2, GPX‐1 and GAPDH. Semi‐quantification of NOX4 level (F), the P‐ERK1/2 density/T‐ERK1/2 density ratio (G), and GPX‐1 level (H). The expression levels of target proteins were normalized to those of GAPDH Data are represented as mean ± standard error of mean (n = 6; *P < .05, **P < .001 vs. IR). CON, sham‐operated control; IR, ischaemia‐reperfusion; NRG‐1, IR + NRG‐1

NOX4 plays an important role in regulating the ROS levels in the myocardial tissue.^28^ The mRNA expression levels of *Nox4* were up‐regulated in the IR group (Figure 3A). Treatment with NRG‐1 significantly mitigated the IR‐induced up‐regulated mRNA levels of *Nox4* (Figure 3A; *P* < .05). Additionally, the expression levels of the antioxidant genes *Cat* and *Gpx‐1* were also examined. IR did not affect the mRNA levels of *Gpx‐1*. In contrast, treatment with NRG‐1 significantly up‐regulated the mRNA levels of *Gpx‐1* (Figure 3B, *P*< .01). The mRNA levels of *Cat* were not significantly different among the three groups (Figure 3C). Immunohistochemical analysis revealed that the level of NOX4 was markedly up‐regulated in the IR group (Figure 3D). The level of NOX4 in the NRG‐1 group was markedly lower than that in the IR group (Figure 3D).

### 3.3 NRG‐1 reduced the expression of NOX4 via the activation of ERK1/2

Phosphorylation of ERK1/2 (44 kDa/42 kDa) is reported to play a critical role in the downstream signals of NRG‐1/ErbB4.^11^ Compared with that in the IR group, the ERK1/2 phosphorylation level was higher in the NRG‐1 group (Figure 3E and G, *P* < .05). The effect of NRG‐1 on the expression levels of NOX4 and GPX‐1 was examined using Western blotting. The expression level of NOX4 in the IR group was significantly higher than that in the CON group. Treatment with NRG‐1 significantly mitigated the IR‐induced up‐regulated NOX4 levels (Figure 3E and F; *P* < .01). NRG‐1 markedly increased the mRNA levels of *Gpx‐1*. However, the levels of GPX‐1 were not significantly different among the three groups (Figure 3E and H; *P* > .05).

### 3.4 NRG‐1 suppressed the NLRP3/caspase‐1 pathway

**FIGURE 4:**
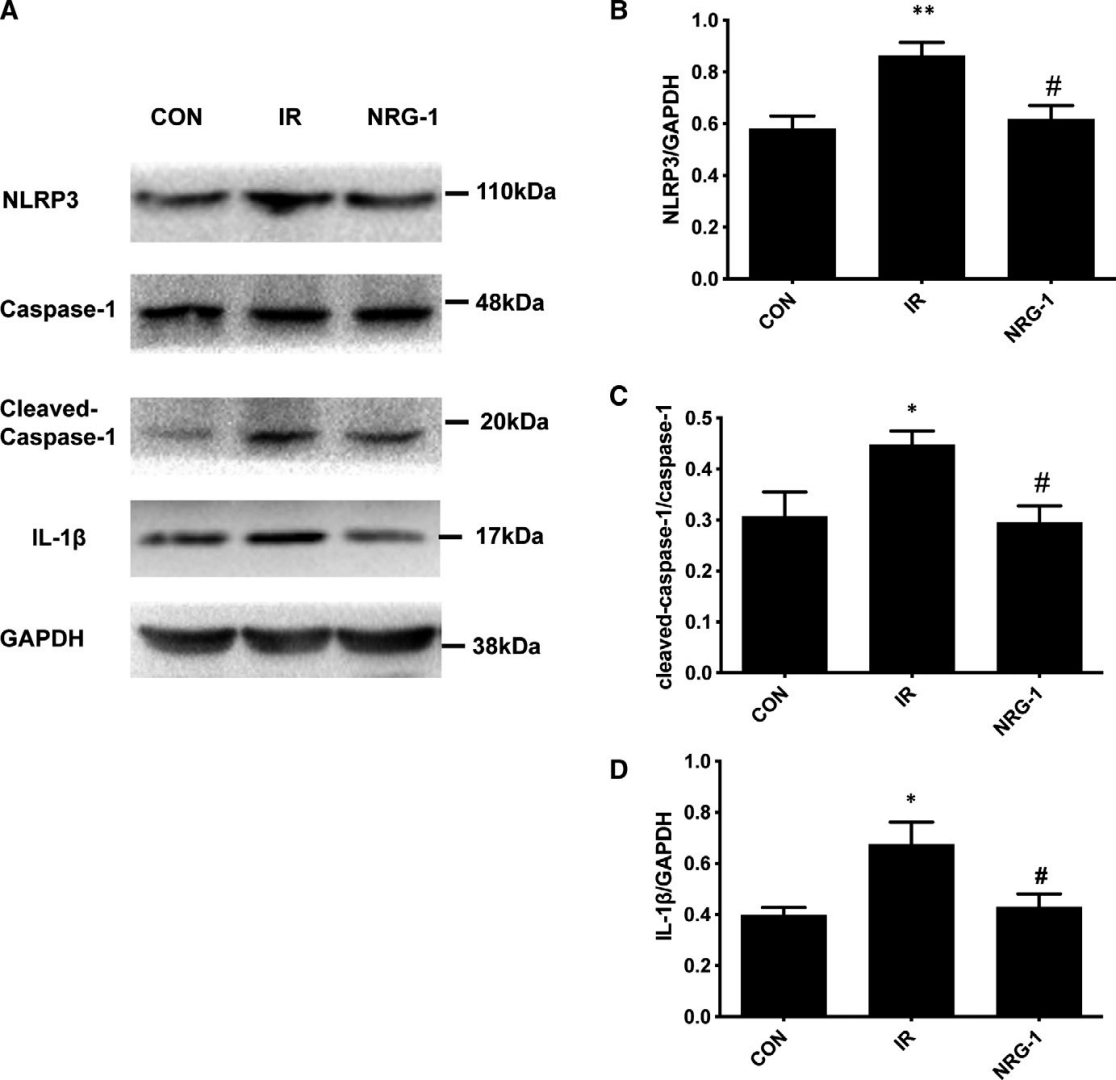
NRG‐1 suppresses the NLRP3/caspase‐1 pathway. A. Representative Western blots showing the levels of NLRP3, caspase‐1, cleaved caspase‐1, IL‐1β and GAPDH. Semi‐quantification of NLRP3 level (B), the cleaved caspase‐1 density/caspase‐1 density ratio (C), and IL‐1β level (D). The expression levels of the target proteins were normalized to those of GAPDH. Data are represented as mean ± standard error of mean (n = 6; *P < .05,**P < .001 vs CON; #*P* < .05 vs. IR). CON, sham‐operated control; IR, ischaemia‐reperfusion; NRG‐1, IR + NRG‐1

The cardioprotective effect of NRG‐1 was evaluated by examining the expression of proteins involved in the NLRP3/caspase‐1 pathway using Western blotting. Compared with that in the CON group, the expression of NLRP3(110 kDa) was up‐regulated in the IR group (Figure 4A‐B; *P* < .01). Treatment with NRG‐1 significantly mitigated the IR‐induced up‐regulated levels of NLRP3 (Figure 4A‐B; *P* < .05). The expression levels of pro‐caspase‐1 (48 kDa) and its activated fragment cleaved caspase‐1 (20 kDa) were determined using Western blotting (Figure 4A). Compared with that in the CON group, the level of cleaved caspase‐1 was significantly up‐regulated in the IR group (Figure 4C; *P* < .05). The expression level of cleaved caspase‐1 in the NRG‐1 group was lower than that in the IR group (Figure 4C; *P* < .05). However, the protein levels of pro‐caspase‐1 were not significantly different among the three groups.IL‐1β, the downstream factor of caspase‐1, was up‐regulated in the IR group at protein level compared with that in the CON group(Figure 4C; *P* < .05). Treatment with NRG‐1 significantly mitigated the NLRP3‐induced up‐regulated levels of IL‐1β (Figure 4A and D; *P* < .05).

### 3.5 PD98059 (PD) inhibited the protective effect of rhNRG‐1 in vitro

**FIGURE 5:**
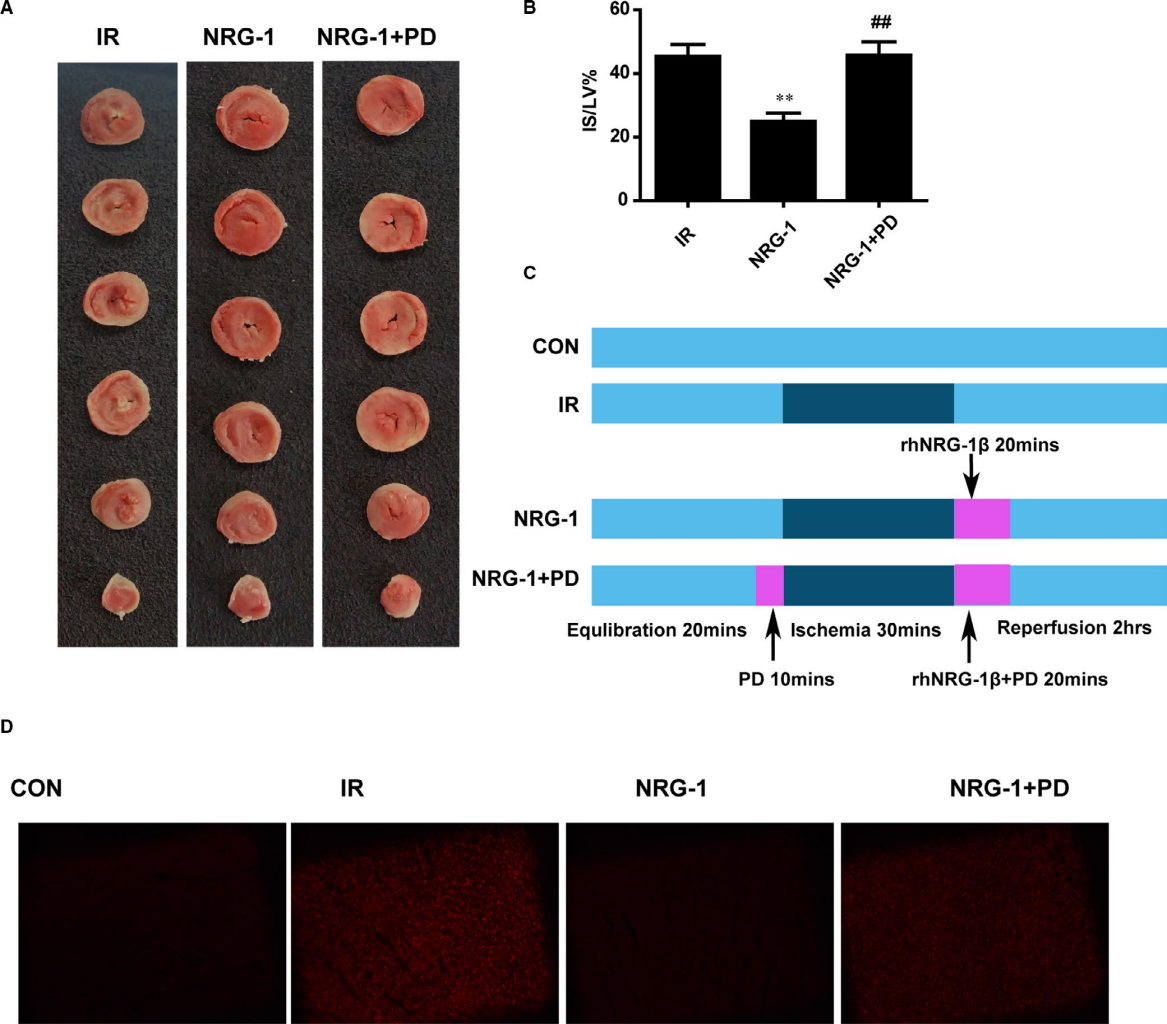
PD98059 mitigates the NRG‐1‐mediated alleviation of myocardial injury. A. Triphenyl tetrazolium chloride (TTC) staining was used to detect the infarct size. Representative cardiac sections stained with TTC (red, ischaemic area; white, infarct area). B. The percentage of infarct size/left ventricle (IS/LV%). C. The Langendorff experiment protocol. D. Representative frozen cardiac sections subjected to dihydroethidium(DHE) staining to detect reactive oxygen species at the risk area. Data are represented as mean ± standard error of mean (n = 6; **P < .01 vs IR; ##*P* < .01 vs. NRG‐1). CON, sham‐operated control; IR, ischaemia‐reperfusion; NRG‐1, IR + NRG‐1; PD, PD98059

**FIGURE 6:**
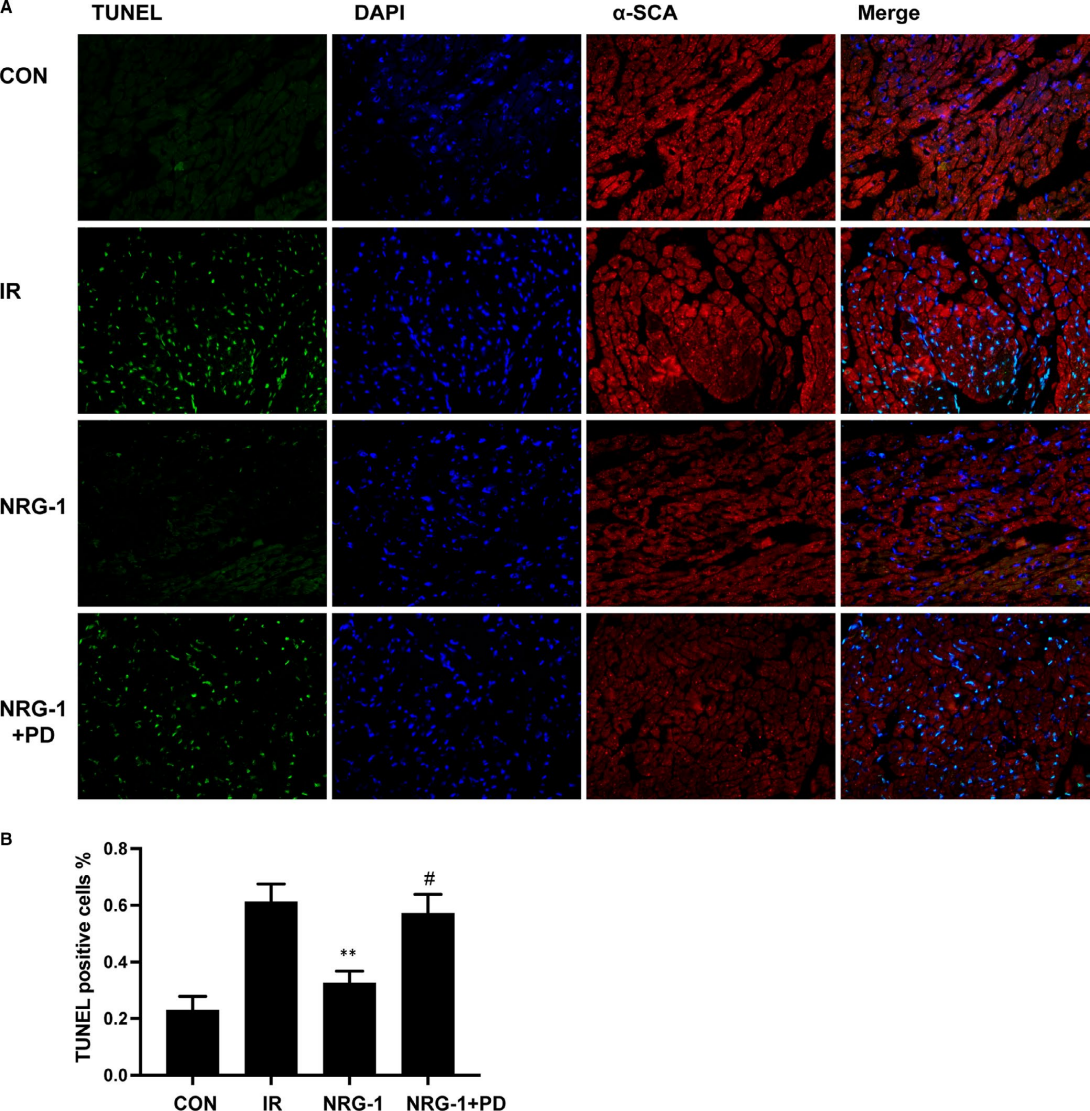
PD98059 increases the number of terminal deoxynucleotidyl transferase dUTP nick end labelling (TUNEL)‐positive cells. (A) Representative myocardial sections subjected to the TUNEL assay. TUNEL‐positive cells are shown in green. The 4′,6‐diamidino‐2‐phenylindole (DAPI)‐stained nuclei are shown in blue. The α‐SCA‐positive cardiomyocytes are shown in red. (B) The percentage of TUNEL‐positive cells among the total number of cells. Data are represented as mean ± standard error of mean (n = 6; **P < .01 vs IR; #*P* < .05 vs. NRG‐1). CON, sham‐operated control; IR, ischaemia‐reperfusion; NRG‐1, IR + NRG‐1; PD, PD98059

To determine the role of ERK1/2 activation in mediating the antioxidant effect of NRG‐1, PD (a MEK inhibitor) was used to inhibit the phosphorylation of ERK1/2 in the Langendorff IR model. Treatment with PD inhibited the protective effect of NRG‐1. The IS value in the NRG‐1 + PD group was higher than that in the NRG‐1 group (Figure 5A–B, *P* < .01). The results of the DHE assay revealed that IR increased ROS production, which was markedly inhibited upon treatment with NRG‐1 (Figure 5D). Treatment with PD inhibited the antioxidant effect of NRG‐1 and consequently enhanced ROS production (Figure 5D). The TUNEL assay was also performed to examine apoptosis in the Langendorff model. The percentage of TUNEL‐positive cells in the NRG‐1 group was significantly lower than that in the IR group (Figure 6A and B; *P* < .01). Compared with that in the NRG‐1 group, the percentage of TUNEL‐positive cells was significantly higher in the NRG‐1 + PD group (Figure 6A and B ; *P* < .05).

### 3.6 PD up‐regulates the expression of NOX4 and NLRP3/caspase‐1 pathway in vitro

**FIGURE 7:**
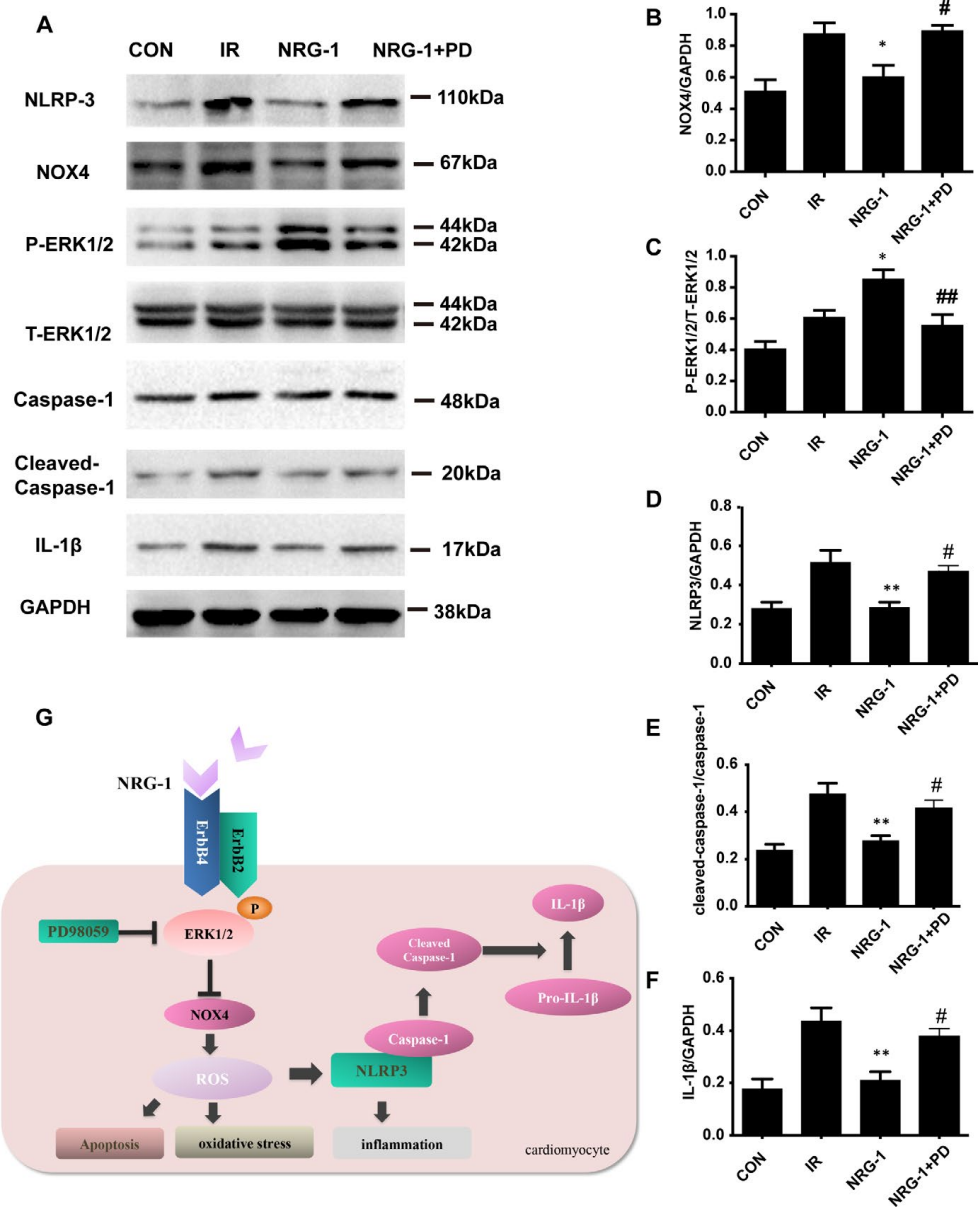
PD98059 up‐regulates the expression of NOX4 and NLRP3/caspase‐1 pathway. A, Representative Western blots showing the levels of NOX4, P‐ERK1/2, T‐ERK1/2, caspase‐1, NLRP‐3, cleaved caspase‐1, IL‐1β and GAPDH. Semi‐quantification of NOX4 level (B), the P‐ERK1/2 density/T‐ERK1/2 density ratio (C), NLRP3 level (D), the cleaved caspase‐1 density/caspase‐1 density ratio (C) and IL‐1β level (E). The expression levels of target proteins were normalized to those of GAPDH. G. A proposed schematic diagram of the molecular pathway activated by NRG‐1. Data are represented as mean ± standard error of mean (n = 6; *P < .05 vs IR; ##*P* < .001 vs. NRG‐1). CON, sham‐operated control; IR, ischaemia‐reperfusion; NRG‐1, IR + NRG‐1; PD, PD98059

To examine the role of ERK1/2 activation in mediating NRG‐1‐induced suppression of NOX4 , PD was used to block the phosphorylation of ERK1/2 in the Langendorff IR model. Treatment with PD markedly inhibited the NRG‐1‐induced phosphorylation of ERK1/2 (Figure 7A and C). Additionally, the expression level of NOX4 in the NRG‐1 group was significantly lower than that in the IR group. Treatment with PD significantly up‐regulated the levels of NOX4 (Figure 7A and B; *P* < .05). The expression of NLRP3, a downstream signal of NOX4, was examined as it is reported to be down‐regulated by NRG‐1 in vivo. In the Langendorff model, NRG‐1 could suppress the expression of NLRP3 (Figure 6 A and D, *P* < .01), which was mitigated upon treatment with PD (Figure 6 A andD,*P* < .05). For downstream factors, the expression levels of pro‐caspase‐1, cleaved caspase‐1 and IL‐1β were determined using Western blotting (Figure 7A). The expression level of cleaved caspase‐1 in the NRG‐1 group was lower than that in the IR group (Figure 7E, *P* < .01). When treated with PD, the cleaved caspase‐1 was significantly up‐regulated compared with the NRG‐1 group(Figure 7E, *P* < .05). The protein levels of pro‐caspase‐1 were not significantly different among the four groups.IL‐1β, the downstream factor of caspase‐1, was down‐regulated in the NRG‐1 group at protein level compared with that in the IR group(Figure 7F; *P* < .01). Treatment with PD significantly up‐regulated the levels of IL‐1β (Figure 7A and F; *P* < .05).

## 4 DISCUSSION

Previously, we had demonstrated that NRG‐1 can down‐regulate apoptosis in cardiomyocytes through the RISK pathway.^16^ Additionally, NRG‐1 is reported to exert an anti‐inflammatory effect in the brain tissue.^18^ Furthermore, NRG‐1 can reduce myocardial oxidative damage by regulating eNOS activity.^29^ In this study, NRG‐1 decreased ROS production through novel pathways and alleviated inflammation‐induced damage. This indicated that NRG‐1 can decrease ROS production by inhibiting NOX4 through ERK1/2 activation and consequently exert protective effects on the myocardial tissue by inhibiting the NLRP3/caspase‐1 pathway.

To define the cardioprotective effects of NRG‐1, we detected the myocardial apoptosis by a TUNEL assay, and α‐SCA immunofluorescence staining was performed to specify the cardiomyocytes. In the present study, we found that NRG‐1 can significantly alleviate apoptosis of myocardial sections both in vivo and in vitro(Figures 2 and 6), which is consistent to previous work.^16^, ^30^ To determine the myocardial injury, plasma CK‐MB was detected. In NRG‐1 group the concentration of CK‐MB was lower than that in IR group, indicating the cardioprotective effects of NRG‐1(Figure 1D).To determine the effect of NRG‐1 on cardiac function, echocardiogram was used to assess the cardiac function. In IR group, the EF% and FS% are both deceased compared with the CON group. This result is consistent to a previous study.^26^However, no significant differences were found between the IR group and NRG‐1 group(Figure 1F‐H).This result indicated that the cardioprotection of NRG‐1 did not rely on the improvement of cardiac function, and its anti‐apoptosis and antioxidant effect will play more important roles in the mechanism of cardioprotection. A previous study showed that long‐term treatment with NRG‐1 would improve cardiac function of the ischaemic heart via anti‐remodelling effects.^26^In our experiment, only a bolus of NRG‐1(3 μg/kg) injected intravenously at the onset of reperfusion was not sufficient to improve cardiac function. We only used EF% and FS% to assess the effect of NRG‐1 on systolic function, the influence of NRG‐1 on the diastolic function in MIRI rats would be examined in the future.

The production of ROS, which is up‐regulated during MIRI, may damage the myocardial tissue through various pathways, including degradation of various structural proteins and enzymes, DNA damage, calcium overload and induction of cardiomyocyte apoptosis.^31^ The levels of 4HNE, which is the product of lipid peroxidation, indicate the levels of ROS production. DHE staining can also determine the content of ROS in the cardiomyocytes. In this study, treatment with NRG‐1 decreased the content of 4HNE in the rat model of MIRI (Figure 1D) and the fluorescence intensity of DHE in the Langendorff model. This indicated that NRG‐1 exerts anti‐inflammatory effects (Figure 5C).

Previous studies have demonstrated that GPX‐1 and CAT are involved in the regulation of ROS clearance in the cardiomyocytes.^32^ To evaluate the effect of NRG‐1 on ROS clearance, the effect of NRG‐1 on the expression of GPX‐1 and CAT was examined. Treatment with NRG‐1 significantly up‐regulated the mRNA expression level of *Gpx‐1* but not that of *Cat* (Figure 2B–C). Therefore, we speculate that NRG‐1 may clear ROS through GPX‐1. However, treatment with NRG‐1 did not affect the expression level of GPX‐1 (Figure 3A and D). There is no evidence for the NRG‐1‐mediated clearance of ROS through GPX‐1, which must be examined in future studies.

The production of intracellular ROS is mediated mainly through NADPH oxidase and the mitochondrial pathway. Recent studies have reported that NOX2 and NOX4, which are the main sources of cardiac ROS, play a vital role in the growth and death of cardiomyocytes.^28^, ^33^ The cardiac tissue‐specific knockout of *Nox4* exacerbates MIRI.^34^ In this study, the effect of NRG‐1 on the expression of NOX4 was examined. NRG‐1 down‐regulated the mRNA expression of *Nox4*. This suggested that the antioxidant effect of NRG‐1 is mediated through the inhibition of NOX4, which leads to the down‐regulation of ROS synthesis. Western blotting and immunohistochemical analyses revealed that NRG‐1 significantly down‐regulated the expression of NOX4 (Figures 2D and 3A). This further confirms that NRG‐1 down‐regulates ROS synthesis by inhibiting NOX4. A previous study found that inhibiting NOX4 may aggravate endothelium dysfunction.^35^In chronically overloaded heart, myocardial NOX4 preserves capillary density.^36^ In the present study, we found that the myocardial capillary density in IR group is lower than that in the CON group which is consistent to a previous study^37^ (Figure 3). But no significant differences were found between the IR group and NRG‐1group (Figure 3).This results demonstrated that a bolus injection of NRG‐1 was not sufficient to affect the capillary density. Whether the effects of NRG‐1 on capillary density was related to the expression of NOX4 remains unknown and more work should be done in the future.

In our previous work, we had proved that the cardioprotective effect of NRG‐1 was dependent on the activation of ErbB4 and the downstream signal ERK1/2.^16^It has been reported that ErbB2 and ErbB4 are expressed in adult rat hearts and ErbB3 is expressed in neonatal rat hearts.^11^But a recent study showed that postconditioning may affect the ROS production and regulate the expression of ErbB3 in MIRI model.^38^This inferred that in our experiment, the ErbB3 may be involved with antioxidant effect of NRG‐1. And the relationship between ErbB3 and NOX4, even the NLRP3/caspase‐1 signal, will be a promising field to explore further.

Next, the upstream and downstream mechanisms of NOX4 regulation were examined. Previous studies have demonstrated that NRG‐1 can activate the ERK1/2 pathway.^11^ The activation of the ERK1/2 pathway is reported to down‐regulate the expression of NOX4,^17^ which results in decreased ROS production. Previously, we had demonstrated that the administration of NRG‐1 can significantly activate ERK1/2 in the MIRI model.^16^ In this study, we demonstrated the negative correlation between NRG‐1 administration and NOX4 expression. Thus, we hypothesized that the inhibitory effect of NRG‐1 on NOX4 expression in the MIRI model depends on the activation of the ERK1/2 pathway. To verify this hypothesis, an ERK1/2 inhibitor (PD98059) was used to verify the correlation between ERK1/2 and NOX4 in the Langendorff model. Treatment with PD98059 blocked ERK1/2 and up‐regulated NOX4 expression. This indicated that PD98059 significantly mitigated the NRG‐1‐mediated down‐regulation of NOX4. These findings suggest that NRG‐1 inhibits NOX4 by activating ERK1/2 and consequently down‐regulating ROS production.

Enhanced levels of ROS can promote cardiomyocyte apoptosis.^28^ Previously, we had reported that NRG‐1 can inhibit cardiomyocyte apoptosis. In addition to promoting cardiomyocyte apoptosis, ROS can damage cardiomyocytes by activating the NLRP3 inflammasome. Inflammation injury also plays an important role in MIRI.^39^ Previous studies have reported the anti‐inflammatory effect of NRG‐1 in cerebral IR injury.^40^ The anti‐inflammatory effect of NRG‐1 in myocardial tissues has not been explored. As NRG‐1 down‐regulated ROS synthesis in this study, we focused on ROS‐mediated NLRP3 activation. Treatment with NRG‐1 significantly down‐regulated the expression of Nlrp3 in vivo and the expression of caspase‐1 and IL‐1β (Figures 4 and 7). This indicated that NRG‐1 exerts myocardial protective effects by down‐regulating NLRP3/caspase‐1 signalling pathway.

The upstream and downstream mechanisms of ERK1/2 and NLRP3 were clarified by examining the expression of NLRP3 in the Langendorff model. Treatment with PD98059 up‐regulated the expression of NLRP3, cleaved caspase‐1 and IL‐1β(Figure 7). This suggested that the expression of NLRP3 is dependent on the NRG‐1‐mediated activation of ERK1/2. The results of this study and previous studies suggest that the activity of NLRP3 is associated with NOX4‐mediated ROS production.

Above all, we conclude that NRG‐1 reduces ROS synthesis by inhibiting NOX4 through ERK1/2, inhibits NLRP3 inflammasome and ultimately plays a role in myocardial protection.

## CONFLICT OF INTEREST

The authors confirm that there are no conflicts of interest.

## AUTHOR CONTRIBUTIONS

**Fuhua Wang:** Conceptualization (lead); Data curation (lead); Formal analysis (lead); Funding acquisition (supporting); Investigation (lead); Methodology (lead); Project administration (supporting); Resources (supporting); Software (lead); Supervision (equal); Validation (lead); Visualization (lead); Writing‐original draft (lead); Writing‐review & editing (lead). **Huan Wang:** Conceptualization (supporting); Data curation (supporting); Methodology (supporting); Validation (supporting); Writing‐review & editing (supporting). **Xuejing Liu:** Data curation (supporting); Formal analysis (supporting); Methodology (supporting); Writing‐review & editing (supporting). **Haiyi Yu:** Methodology (supporting); Supervision (supporting); Writing‐review & editing (supporting). **Xiaomin Huang:** Data curation (supporting); Methodology (supporting); Validation (supporting); Writing‐review & editing (supporting). **Wei Huang:** Conceptualization (equal); Funding acquisition (equal); Project administration (equal); Resources (equal); Supervision (equal); Validation (equal); Writing‐review & editing (equal). **Guisong Wang:** Conceptualization (equal); Funding acquisition (lead); Project administration (equal); Supervision (equal); Writing‐review & editing (equal).

## Data Availability

The data that support the findings of this study are available from the corresponding author upon reasonable request.
